# Impact of maternal risk factors on ethnic disparities in maternal mortality: a national population-based cohort study

**DOI:** 10.1016/j.lanepe.2024.100893

**Published:** 2024-03-29

**Authors:** Nicola Vousden, Kathryn Bunch, Sara Kenyon, Jennifer J. Kurinczuk, Marian Knight

**Affiliations:** a NIHR Policy Research Unit in Maternal and Neonatal Health and Care, National Perinatal Epidemiology Unit, Nuffield Department of Population Health, University of Oxford, UK; b Institute of Applied Health Research, University of Birmingham, UK

**Keywords:** Maternal mortality, Ethnicity, Inequality, Socioeconomic deprivation

## Abstract

**Background:**

Ethnic disparities in maternal mortality are consistently reported. This study aimed to investigate the contribution of known risk factors including age, socioeconomic status, and medical comorbidities to observed ethnic disparities in the United Kingdom (UK).

**Methods:**

A cohort of all women who died during or up to six weeks after pregnancy in the UK 2009–2019 were identified through national surveillance. No single denominator population included data on all risk factors, therefore we used logistic regression modelling to compare to 1) routine population birth and demographic data (2015–19) (routine data comparator) and 2) combined control groups of four UK Obstetric Surveillance System studies (UKOSS) control comparator)).

**Findings:**

There were 801 maternal deaths in the UK between 2009 and 2019 (White: 70%, Asian: 13%, Black: 12%, Chinese/Other: 3%, Mixed: 2%). Using the routine data comparator (n = 3,519,931 maternities) to adjust for demographics, including social deprivation, women of Black ethnicity remained at significantly increased risk of maternal death compared with women of white ethnicity (adjusted OR 2.43 (95% Confidence Interval 1.92–3.08)). The risk was greatest in women of Caribbean ethnicity (aOR 3.55 (2.30–5.48)). Among women of White ethnicity, risk of mortality increased as deprivation increased, but women of Black ethnicity had greater risk irrespective of deprivation. Using the UKOSS control comparator (n = 2210), after multiple adjustments including smoking, body mass index, and comorbidities, women of Black and Asian ethnicity remained at increased risk (aOR 3.13 (2.21–4.43) and 1.57 (1.16–2.12) respectively).

**Interpretation:**

Known risk factors do not fully explain ethnic disparities in maternal mortality. The impact of socioeconomic deprivation appears to differ between ethnic groups.

**Funding:**

This research is funded by the 10.13039/501100000272National Institute for Health and Care Research (NIHR) Policy Research Programme, conducted through the Policy Research Unit in Maternal and Neonatal Health and Care, PR-PRU-127-21202.

**Research in context**

**Evidence before this study**

Confidential enquiry into deaths of women during pregnancy or up to a year after the end of pregnancy between 2000 and 2023 shows evidence of higher maternal mortality amongst women from Black and Asian aggregated ethnic groups compared with women from White ethnic groups. Similar disparities have been identified in the US. The factors associated with increased risk of maternal death are widely established, including maternal age, socioeconomic status and underlying medical conditions. We searched PubMED for peer reviewed papers published from inception to 01/03/2024 using the terms preg∗ OR matern∗ AND ethnicity OR racial groups-statistics and numerical data AND maternal mortality AND socioeconomic factors. One national study using historical data and four regional or single centre studies in the USA report that maternal age, socioeconomic status, underlying medical conditions and antenatal care all contribute to ethnic disparities in maternal mortality, but that disparities in risk between ethnic groups remain even after taking account of these. To our knowledge, there is no evidence that describes the extent to which these factors contribute to the increased risk of death amongst different ethnic groups in the UK. In addition, most current estimates of risk from national surveillance reports are also limited to aggregate ethnic groups due to the lack of availability of detailed denominator data.

**Added value of this study**

This study used both routine population and research data to determine the extent to which known risk factors for maternal mortality contribute to disparities in maternal death observed between ethnic groups. The large number of events in this 11-year national cohort, and the use of two denominator data sources, meant that multiple adjustment for known risk factors was possible, including in detailed ethnic groups. Therefore, for the first time we can conclude that women of Black ethnicity remained at 3.3-fold (330%) increased risk of maternal death compared with women of white ethnicity after multiple adjustment including for smoking, body mass index, and medical comorbidities and at 2.4-fold (240%) increased risk after adjusting for demographic factors, including social deprivation. The risk was greatest in women of Caribbean ethnic origin. After multiple adjustment including smoking, body mass index, and medical comorbidities, women of Asian ethnicity were at 0.57 fold (57%) increased risk of maternal death overall. However, after adjusting for deprivation, only women of Indian ethnic origin remained at significantly increased risk. To our knowledge this is the first study to describe the varying impact of socioeconomic deprivation on maternal mortality within ethnic groups in the UK. Among women of White ethnicity, risk of mortality increased as deprivation increased, but in women of Black ethnicity this gradient was not observed and risk was greater irrespective of deprivation.

**Implications of all the available evidence**

Whilst age, BMI, socioeconomic status, and pre-existing medical complications are important risks for maternal mortality in the UK, we demonstrate that ethnicity remains an important independent risk. Policy and practice should aim to optimise pre-pregnancy health but also move beyond addressing individual factors to improving equity in underlying structures such as housing, education, and access to healthy environments. Efforts to tackle implicit structural bias and deliver culturally competent maternity care are vital to reduce the existing inequalities between ethnic groups. Detailed understanding of how to adapt and deliver culturally competent services and policies is required.

## Introduction

For over 20 years, the UK Confidential Enquiry into Maternal Deaths and the USA Pregnancy Mortality Surveillance System have consistently shown that women of Black, Asian^1^^,^^2^ and American Indian and Alaska Native ethnic origin^3^^,^^4^ experience significantly increased risk of dying while pregnant or within six weeks of the end of pregnancy, when compared with women of White ethnic origin. In 2019–2021 in the UK, women of Black ethnic origin were at 3.8 times greater risk, and Asian women at 1.8 times greater risk than women of White ethnic origin.^2^ This has led to widespread national policy actions, such as the establishment of the governmental Maternity Disparities Taskforce in the UK^5^ and the Alliance for Innovation on Maternal Health in the USA,^6^ aiming to improve clinical outcomes and care experiences for women from ethnic minority groups. However, policy change is hindered by a lack of clarity about the underlying causes of disparities relating to ethnicity.

The pathways that contribute to increased risk of maternal mortality are complex and interrelated. For example, wider structural and cultural factors such as education, employment, and health beliefs inform the communities in which we live, access to health care services, and our individual health risks such as obesity, underlying medical conditions and age at pregnancy.^7^ The impact of ethnicity and structural racism are deeply embedded across all levels.^8^ Evidence from the USA suggests that there are differences in cause of maternal death depending upon ethnicity,^9^ for example women of Black ethnicity are significantly more likely to die from cardiomyopathy, pulmonary embolism and hypertensive disorders of pregnancy compared with women of White ethnicity.^3^ However, in the UK, whilst pre-existing medical comorbidities have been shown to significantly increase the risk of maternal death,^10^ and women from South Asian and Black ethnic groups are known to have higher rates of hypertension and diabetes,^11^ ethnic disparities in cause of death have not been observed.^12^ In addition, women living in the most deprived areas have a two fold greater risk of maternal mortality than those in the least deprived areas (RR 2.03, 95% CI 1.25–3.43).^2^ Since a greater proportion of women of Black ethnicity live in the most deprived areas^13^ this could contribute to observed ethnic disparities. Differing access to, and utilisation of, antenatal care are also known to be associated with ethnicity so are hypothesised to contribute.^8^^,^^14^ Furthermore, women from Black and Asian ethnicity are more likely to be multiparous^14^ and therefore hypothesised to be at risk of poorer maternal outcomes, however in the UK multiparity has been shown to independently increase the risk of severe morbidity^14^^,^^15^ but not maternal death.

There is limited evidence from the USA that whilst maternal age, socioeconomic status, underlying medical conditions and receipt or quality of antenatal care all contribute to ethnic disparities in maternal mortality, even after taking account of these, women of Black ethnicity remain at increased risk of maternal mortality than women of White ethnicity.^16^, ^17^, ^18^, ^19^, ^20^ Whilst ethnic disparities in maternal mortality are widely reported in the UK, the extent to which known associated factors such as socioeconomic deprivation, parity and underlying medical conditions contribute is not known since crude rates by ethnic group are usually presented.^2^ Therefore, the aim of this study was to use population level data from both routine and research sources to investigate the contribution of known risk factors including age, socioeconomic status, and medical comorbidities to observed ethnic disparities in maternal mortality in the UK.

## Methods

### Women who died

Socio-demographic, medical and pregnancy related characteristics of all women who died from direct or indirect causes during or up to six weeks after the end of pregnancy in the UK between 2009 and 2019 were extracted from MBBRACE-UK records. MBRRACE-UK is the organisation responsible for enhanced maternal death surveillance in the UK which holds a database of every maternal death in the UK since 2009, cross-checked with linked vital statistics records.^2^ Women's ethnicity was self-reported in their maternity records and classified according to five aggregate, or high-level, groups (Asian, Black, Mixed or Multiple, Other or White) and 18 sub-categories as used in the 2021 census classification for England and Wales (listed in Supplementary Table S1).^21^^,^^22^ Ethnicity in this study is therefore defined as the ethnic group that the woman feels they belong to, which could be based on their culture, family background, identity or physical appearance for consistency with other UK data and self-report methods.^23^ Prior to 2011 the census classified Chinese ethnic group as ‘Other’ ethnic group, not ‘Asian’, therefore this classification is retained in this study. The terms ‘pregnant women’ and ‘mothers’ will be used throughout this paper to reflect the recorded characteristics of individuals identified in the MBRRACE case notes, but the authors recognise not everyone who is pregnant or giving birth will identify as a woman or a mother. Socio-economic status was measured by quintiles of the area-based Index of Multiple Deprivation (IMD). These were assigned at the points when records were prepared for routine annual reporting.

### Comparison data

No single denominator population could provide information on all additional factors potentially contributing to maternal death. For example, publicly available routine vital statistics data on women giving birth in England does not include information about factors such as smoking, body mass index (BMI), medical or pregnancy history.

#### Routine population data

Anonymised information about individual births in the UK between 2015 and 2019 was extracted from various sources: the Office for National Statistics, Information Services Division (Scotland), the Northern Ireland Regional Maternity System, the NHS England Personal Demographic Service and the NHS Number for Babies Service and combined into a single dataset. Records relating to births in Jersey and Guernsey were also extracted but none of these records included postcode, meaning that no measure of socio-economic deprivation could be derived and thus these records could not contribute to the analyses.

#### UKOSS control data

While the population comparator data described above enabled adjustment for various socio-demographic and pregnancy related characteristics including socio-economic status, it did not include any information about the individual women's medical characteristics. A second comparator data set was assembled from control data previously collected for four separate United Kingdom Obstetric Surveillance System (UKOSS) studies on Seasonal Influenza (n = 694), Haemolysis, Elevated liver enzymes and Low platelet count (HELLP) (n = 488), Placenta Accreta (n = 266) and Sepsis (n = 762).^24^ The UKOSS methodology is described in detail elsewhere.^25^ Non-identifiable data are collected from all 194 consultant-led obstetric units in the UK each month, with a rigorous process to ensure a high level of data completeness. Participating hospitals selected control women as those who gave birth immediately before the index case with the condition under surveillance.

### Details of ethics approval

Identifiable MBRRACE-UK data were collected in England and Wales without consent with approval of the Secretary of State for Health and Social Care under Section 251 of the NHS Act 2006 (15/CAG/0119). Data were collected in Scotland without consent with approval from the Public Benefit and Privacy Panel for Health and Social Care (1920–0131). Identifiable information was not provided from Northern Ireland. The legal basis for this activity is Article 6 (1) (e) and Article 9 (2) (i) under the General Data Protection Regulation. Identifiable data were then de-identified prior to this analysis. Each UKOSS study had ethical approval by the North London REC1 (reference number: 10/H0717/20).

### Statistical analysis

All analyses were carried out using Stata 17. Statistical significance was assumed to be a P value of less than 0.05. For each of the two comparator datasets, unconditional logic regression was undertaken to estimate the association of ethnicity and other independent variables with maternal death, the primary outcome as identified from the MBRRACE-UK enhanced surveillance. A Directed Acyclic Graph (DAG, Supplementary Table S1) was used to conceptually represent the association of covariates based on existing literature and clinical knowledge. All covariates that were measured and thought to be associated with ethnicity or maternal mortality were included in the multivariate modelling.^26^ Since this data reports on all deaths in the UK compared to all births using population data, no sample size estimate was undertaken.

#### Routine population comparison

Covariates in this comparator group were maternal age (categorised in five-year age bands), multiple pregnancy, ethnic group, socioeconomic status and parity. Records relating to multiple births were identified and reduced so that the dataset consisted of a single record per mother (referred to as a maternity). Between 2015 and 2018 ethnicity data was available as five aggregated, or high level, groupings used in the census classification (Asian, Black, Mixed or Multiple, Other or White), which were used in the main analyses presented in Table 1. Detailed ethnic categorisations were available in the routine comparison dataset in 2018/19, therefore these were used in the sub-analyses presented in Table 2, to give more detailed information in disaggregate ethnic groups. Socio-economic status was derived from quintiles of the area-based Index of Multiple Deprivation (IMD) using government data from each of the devolved nations and based on the residential area of the woman at the time of childbirth. The latest available IMD data was used at the time of data processing (2020 for 2018 births, 2021 for all other birth years). There was no information on parity available for women giving birth in 2015 in the routine data. Since parity was identified as a factor potentially associated with both ethnicity and maternal mortality in the DAG, two models were created, model 1: logistic regression with robust standard errors to assess the impact of maternal age, multiple pregnancy, ethnic group and IMD on maternal mortality and model 2: model 1 plus parity and excluding all comparison data from 2015. Where any other covariates were missing, these were reported in the tables and complete case analysis undertaken. Since being born abroad was considered to precede and determine ethnicity in a large proportion of women, it was not included in either model.

**Table 1:** Comparison of women of known ethnicity who died in the UK 2009 to 2019 from direct or indirect causes while pregnant or within 42 days of the end of pregnancy (MBRRACE-UK) and all women giving birth in the UK 2015–2019.

	Women who died (N = 787^a^)	Women giving birth 2015–2019 (N = 3,519,931)	OR (95% CI)	P value	aOR^b^ (95% CI)	P value	aOR^d^ (95% CI)	P value
	Number (%)	Number (%)						
**Socio-demographic characteristics**								
Maternal age								
<20	34 (4)	106,398 (3)	1.73 (1.17–2.57)	0.006	1.78 (1.16–2.73)	0.008	1.81 (1.18–2.79)	**0.007**
20–24	92 (12)	498,641 (14)	1 (ref.)	–	1 (ref.)	–	1 (ref.)	**1 (ref.)**
25–29	187 (24)	960,125 (27)	1.06 (0.82–1.35)	0.671	1.18 (0.90–1.55)	0.225	1.15 (0.88–1.52)	**0.306**
30–34	207 (26)	1,090,955 (31)	1.03 (0.80–1.31)	0.823	1.18 (0.90–1.56)	0.223	1.15 (0.87–1.51)	**0.328**
35–40	192 (24)	613,664 (17)	1.70 (1.32–2.17)	0.000	1.94 (1.47–2.56)	0.000	1.85 (1.40–2.45)	**0.000**
≥40	75 (10)	140,732 (4)	2.89 (2.13–3.92)	0.000	3.12 (2.22–4.38)	0.000	3.02 (2.15–3.25)	**0.000**
Missing	0 (0)	109,416 (3)	–	–	–	–		
Ethnic group								
Asian	100 (13)	359,109 (10)	1.34 (1.08–1.66)	0.007	1.19 (0.94–1.49)	0.150	1.18 (0.94–1.49)	**0.161**
Black	105 (13)	157,206 (4)	3.21 (2.61–3.97)	0.000	2.43 (1.92–3.08)	0.000	2.36 (1.85–3.00)	**0.000**
Chinese/Other	23 (3)	98,296 (3)	1.13 (0.74–1.71)	0.573	0.94 (0.60–1.48)	0.796	0.94 (0.60–1.47)	**0.775**
White	543 (69)	2,616,607 (74)	1 (ref.)		1 (ref.)		1 (ref.)	**1 (ref.)**
Missing	16 (2)^a^	288,713 (8)	–	–	–	–	–	**–**
Social deprivation (Quintiles of IMD)								
I (least deprived)	73 (9)	532,440 (15)	1 (ref.)		1 (ref.)		1 (ref.)	**1 (ref.)**
II	90 (11)	605,708 (17)	1.08 (0.80–1.48)	0.610	1.08 (0.79–1.48)	0.622	1.06 (0.78–1.45)	**0.698**
III	110 (14)	670,699 (19)	1.20 (0.89–1.61)	0.235	1.20 (0.89–1.62)	0.236	1.19 (0.88–1.61)	**0.248**
IV	180 (23)	785,770 (22)	1.67 (1.27–2.19)	0.000	1.65 (1.25–2.18)	0.000	1.63 (1.233–2.15)	**0.001**
V (most deprived)	249 (32)	925,314 (26)	1.96 (1.51–2.55)	0.000	1.88 (1.42–2.48)	0.000	1.85 (1.40–2.45)	**0.000**
Missing	85 (11)	0 (0)	–	–	–	–	–	**–**
Born abroad								
UK born	528 (67)	2,581,103 (73)	1 (ref.)	–	–c	–	–c	**–**
Born abroad	189 (24)	929,036 (26)	0.99 (0.84–1.17)	0.948	–c	–	–c	**–**
Missing	70 (9)	9792 (<1)	–	–	–	–	–	**–**
**Pregnancy related characteristics**								
Parity								
0	301 (38)	1,164,937 (33)	1 (ref.)	–	–	–	1 (ref.)	**1 (ref.)**
≥1	479 (61)	1,617,102 (46)	1.15 (0.99–1.32)	0.063	–	–	1.01 (0.86–1.19)	**0.89**
Missing	7 (1)	737,892 (21)	–	–	–	–	–	**–**
Known multiple pregnancy								
No	765 (97)	3,467,723 (99)	1 (ref.)	–	1 (ref.)	–	1 (ref.)	**1 (ref.)**
Yes	22 (3)	52,208 (1)	1.91 (1.25–2.92)	0.003	1.84 (1.18–2.87)	0.008	1.78 (1.13–2.82)	**0.013**

Abbreviations: IMD, Index Multiple Deprivation, UK, United Kingdom.

a The population dataset provides only the ethnic group of the baby. The ethnic group of mothers of Mixed or Multiple ethnic group babies was therefore uncertain and so women of Mixed or Multiple ethnicity, and those giving birth to Mixed ethnicity babies, were recorded as unknown ethnicity, and excluded from this analysis (n = 14).

b Model 1: Adjusted for Age group, aggregate ethnic group, IMD quintiles, known multiple pregnancy.

c Being born abroad was considered a determinant of ethnicity in some women therefore not included in the multivariable models.

d Model 2: Model 1 plus parity excluding 2015 controls.

**Table 2:** Comparison of women of known ethnicity who died in the UK 2009 to 2019 from direct or indirect causes while pregnant or within 42 days after giving birth (MBRRACE-UK) and all women giving birth in the UK 2018–2019 (a subset of Table 1 data when detailed ethnic categorisations were available).

	Women who died (N = 787^a^)	Women giving birth 2018–2019 (N = 1,351,722)	OR (95% CI)	P value	aOR (95% CI)^b^	P value
	Number (%)	Number (%)				
**Socio-demographic characteristics**						
Age group						
<20	34 (4)	37,461 (3)	1.79 (1.21–2.65)	0.004	1.86 (1.21–2.87)	**0.005**
20–24	92 (12)	181,129 (13)	1 (ref.)		1 (ref.)	
25–29	187 (24)	362,017 (27)	1.02 (0.79–1.31)	0.895	1.13 (0.86–1.48)	**0.85**
30–34	207 (26)	426,474 (32)	0.96 (0.75–1.22)	0.717	1.13 (0.86–1.42)	**0.565**
35–40	192 (24)	243,638 (18)	1.55 (1.21–1.99)	0.001	1.72 (1.30–2.28)	**0.000**
≥40	75 (10)	56,021 (4)	2.64 (1.94–3.58)	0.000	2.80 (1.99–3.95)	**0.000**
Missing	0 (0)	45,032 (3)	–	–	–	**–**
Ethnic group						
Bangladeshi	14 (2)	18,699 (1)	1.38 (0.81–2.34)	0.239	1.19 (0.68–2.08)	**0.545**
Indian	35 (4)	40,112 (3)	1.60 (1.14–2.26)	0.007	1.51 (1.04–2.17)	**0.028**
Pakistani	36 (5)	54,559 (4)	1.21 (0.86–1.70)	0.264	1.08 (0.76–1.54)	**0.669**
Other Asian	15 (2)	26,347 (2)	1.05 (0.63–1.75)	0.864	0.84 (0.47–1.48)	**0.539**
African	72 (9)	42,728 (3)	3.10 (2.42–3.96)	0.000	2.13 (1.60–2.84)	**0.000**
Caribbean	24 (3)	10,180 (1)	4.33 (2.88–6.52)	0.000	3.55 (2.30–5.48)	**0.000**
Other Black	9 (1)	7518 (1)	2.20 (1.14–4.25)	0.019	1.98 (1.02–3.85)	**0.043**
**Chinese/Other**	23 (3)	38,106 (3)	1.11 (0.73–1.68)	0.628	0.93 (0.59–1.46)	**0.758**
White	543 (69)	997,370 (74)	1 (ref.)	–	1 (ref.)	**–**
Missing	16 (2)^a^	116,153 (9)	–	–	–	**–**
Social deprivation (Quintiles of IMD)						
I (least deprived)	73 (9)	205,808 (15)	1 (ref.)	–	1 (ref.)	**–**
II	90 (11)	234,893 (17)	1.08 (0.79–1.47)	0.624	1.06 (0.77–1.44)	**0.725**
III	110 (14)	258,537 (19)	1.20 (0.89–1.61)	0.228	1.19 (0.88–1.60)	**0.263**
IV	180 (23)	300,504 (22)	1.69 (1.29–2.22)	0.000	1.62 (1.23–2.14)	**0.000**
V (most deprived)	249 (32)	352,030 (26)	1.99 (1.54–2.59)	0.000	1.86 (1.41–2.45)	**0.000**
Missing	85 (11)	0 (0)	–	–	–	**–**
Born abroad						
UK born	528 (67)	986,948 (73)	1 (ref.)	–	–c	**–**
Born abroad	189 (24)	360,936 (27)	0.98 (0.83–1.16)	0.800	–c	**–**
Missing	70 (9)	3888 (<1)	–	–	–	**–**
**Pregnancy related characteristics**						
Parity						
0	301 (38)	572,622 (42)	1 (ref.)	–	1 (ref.)	**1 (ref.)**
≥1	479 (61)	775,379 (57)	1.18 (1.02–1.36)	0.028	1.06 (0.90–1.24)	**0.488**
Missing	7 (1)	3771 (<1)	–	–	–	**–**
Known Multiple pregnancy						
No	765 (97)	1,332,248 (99)	1 (ref.)	–	1 (ref.)	**–**
Yes	22 (3)	19,524 (1)	1.96 (1.28–3.00)	0.002	1.89 (1.21–2.95)	**0.005**

Abbreviations: IMD, Index Multiple Deprivation, UK, United Kingdom.

a The population dataset provides only the ethnic group of the baby. The ethnic group of mothers of Mixed or Multiple ethnic group babies was therefore uncertain and so women of Mixed or Multiple ethnicity, and those giving birth to Mixed ethnicity babies, were recorded as unknown ethnicity, and excluded from this analysis (n = 14).

b Model 2: Adjusted for Age group, detailed ethnic group, IMD quintiles, known multiple pregnancy, parity.

c Being born abroad was considered a determinant of ethnicity in some women therefore not included in the multivariable models.

#### UKOSS control comparison

The covariates included in this analysis were: maternal age categorized in five-year age bands, aggregated ethnic group, smoking status, Body Mass Index (BMI: underweight, normal, overweight, obese), known history of epilepsy, hypertension, cardiac disease, diabetes, and any other medical condition and pregnancy related factors (parity, multiple pregnancy, and gestational diabetes) (model 3). Where any data were missing, these were reported in the tables and complete case analysis undertaken except where ethnicity was not stated women were included in the “white European” group because the re-distributed proportions matched with the estimated ethnic profiles in the UK population census. Where necessary binary variables were generated from non-ordered categorical variables to allow the fitting of the multivariable model (e.g., for ethnicity White v. other).

### Role of the funding source

The study sponsor (University of Oxford) and the funder played no role in study design; in the collection, analysis, and interpretation of data; in the writing of the report; or in the decision to submit the paper for publication. MK, KB and NV had access to the surveillance data. NV and MK confirm the authors were not precluded from accessing data in the study and take the responsibility to submit for publication.

## Results

**Fig. 1:**
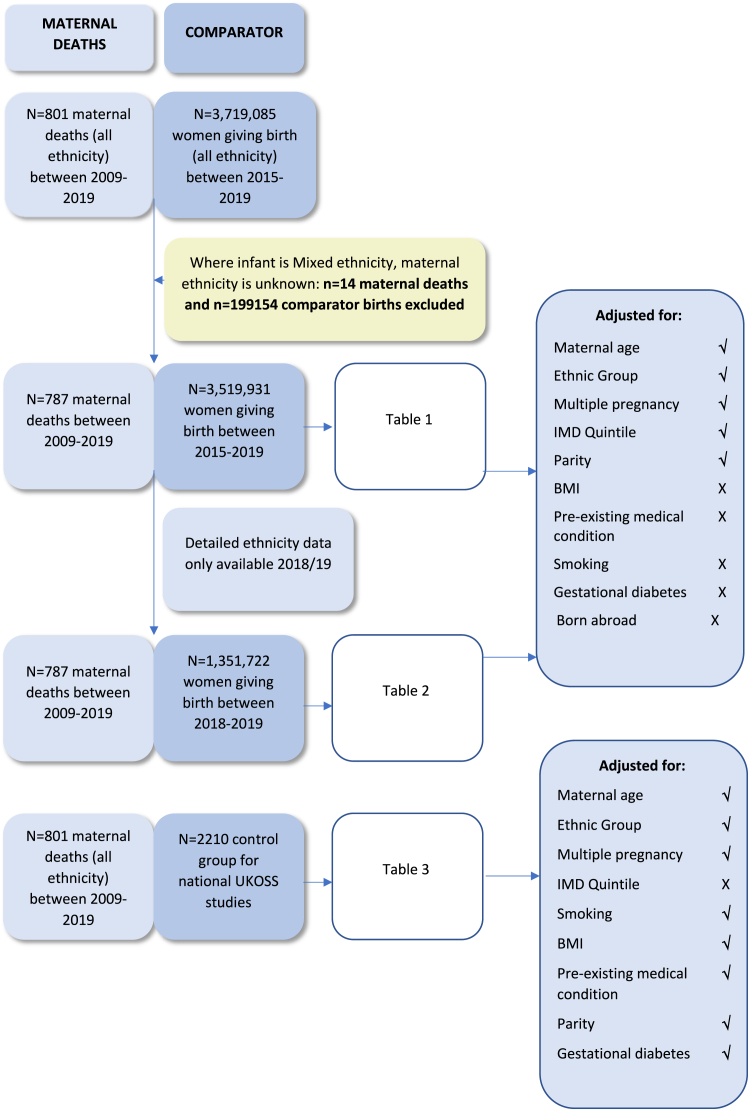
**Flow diagram showing source of comparison data and available variables included in the adjusted models**.

In total 801 women died in the UK between 2009 and 2019 during, or within 6 weeks after the end of, pregnancy, 559 (70%) were in the White aggregated (high-level) group, 100 (12%) were in the Black aggregated group, 105 (13%) were in the Asian aggregated group, 23 (3%) were in the Chinese or Other aggregated group and 14 (2%) were in the Mixed aggregated group. While MBRRACE-UK records include the mother's detailed ethnic group, the routine population dataset provided only the ethnic group of the baby. The ethnic group of mothers of Mixed ethnic group babies was therefore uncertain and so women of Mixed ethnicity, and those giving birth to Mixed ethnicity babies, as shown in Supplementary Table S2, were excluded from further analyses involving population data (n = 14, Tables 1 and 2, Fig. 1), leaving 787 maternal deaths in the analysis using routine population data.

Using the routine population data of all women giving birth in the UK between 2015 and 19 as the comparator group, we found that women from Black and Asian aggregate ethnic groups were at increased risk of maternal mortality compared with women of white ethnicity (OR 3.21 (95% CI 2.61–3.97) and 1.34 (1.08–1.66) respectively) (Table 1). Increasing maternal age, increasing social deprivation, and known multiple pregnancy were all associated with significantly increased risk of maternal death. After adjusting for these three characteristics, women of Black aggregate ethnic origin remained at significantly increased risk of maternal death (aOR 2.43 (1.92–3.08)) compared with women of White ethnicity. The risk of maternal death for women of Asian aggregate ethnicity was increase by 19% but was not statistically significant compared with women of White ethnicity (aOR 1.19 (0.94–1.49)). Adjusting for parity did not substantially change these estimates (Table 1).

In the whole cohort, women aged 40 or over were at significantly increased risk of maternal death in all three adjustment models. Women aged under 20 were at significantly increased risk after adjusting for socioeconomic deprivation (Tables 1 and 2), but not when smoking, BMI and medical comorbidities were considered (Table 3). After adjustment, women that were known to have smoked in pregnancy, that were obese, had known epilepsy, hypertension, cardiac disease, diabetes or other pre-existing medical problem or had a known multiple pregnancy, were at significantly increased risk of maternal death (Table 3). After adjusting for demographics including socioeconomic deprivation, being born abroad was not associated with significantly increased risk (Table 2).

**Table 3:** Characteristics of women who died in the UK 2009 to 2019 from direct or indirect causes while pregnant or within 42 days after giving birth and control women from four UKOSS studies.

	Women who died (N = 801)	UKOSS control women (N = 2210)	OR (95% CI)	P value	aOR^a^ (95% CI)	P value
	Number (%)	Number (%)				
**Socio-demographic characteristics**						
Age group (6 levels)						
<20	35 (4)	119 (5)	1.15 (0.74–1.78)	0.546	1.13 (0.68–1.87)	**0.647**
20–24	94 (12)	366 (17)	1 (ref.)	–	–	**–**
25–29	190 (24)	602 (27)	1.23 (0.93–1.62)	0.148	1.19 (0.86–1.64)	**0.300**
30–34	210 (26)	646 (29)	1.27 (0.96–1.67)	0.093	1.17 (0.85–1.61)	**0.348**
35–40	195 (24)	357 (16)	2.13 (1.60–2.83)	0.000	1.83 (1.31–2.56)	**0.000**
≥40	77 (10)	117 (5)	2.56 (1.78–3.70)	0.000	1.90 (1.23–2.94)	**0.004**
Missing	0 (0)	3 (<1)				
Ethnic group						
Asian	100 (12)	249 (11)	1.28 (0.99–1.64)	0.055	1.57 (1.16–2.12)	**0.004**
Black	105 (13)	106 (5)	3.15 (2.37–4.20)	0.000	3.13 (2.21–4.43)	**0.000**
Chinese/Other	23 (3)	44 (2)	1.66 (1.00–2.78)	0.052	1.76 (0.97–3.19)	**0.061**
Mixed or Multiple	14 (2)	32 (1)	1.39 (0.74–2.63)	0.307	1.08 (0.51–2.29)	**0.837**
White (inc. missing)	559 (70)	1779 (81)	1 (ref.)	–	1 (ref.)	**–**
Known to have smoked during pregnancy						
No	590 (74)	1751 (79)	1 (ref.)	–	1 (ref.)	**–**
Yes	211 (26)	459 (21)	1.36 (1.13–1.65)	0.001	1.57 (1.24–1.97)	**0.000**
**Medical characteristics**						
Bodyweight						
Underweight	13 (2)	43 (2)	0.98 (0.52–1.84)	0.949	0.73 (0.36–1.50)	**0.392**
Normal	321 (40)	1040 (47)	1 (ref.)	–	1 (ref.)	**–**
Overweight	200 (25)	584 (26)	1.11 (0.91–1.36)	0.317	1.02 (0.81–1.30)	**0.838**
Obese	252 (31)	478 (22)	1.71 (1.40–2.08)	0.000	1.46 (1.16–1.85)	**0.001**
Missing	15 (2)	65 (3)				
Known epilepsy						
No	749 (94)	2192 (99)	1 (ref.)	–	1 (ref.)	**–**
Yes	52 (6)	18 (1)	8.45 (4.92–14.54)	0.000	20.98 (11.83–37.23)	**0.000**
Known hypertension						
No	765 (96)	2201 (>99)	1 (ref.)	–	1 (ref.)	**–**
Yes	36 (4)	9 (<1)	11.51 (5.52–24.00)	0.000	14.95 (6.79–32.93)	**0.000**
Known cardiac disease						
No	754 (94)	2188 (99)	1 (ref.)	–	1 (ref.)	**–**
Yes	47 (6)	22 (1)	6.20 (3.71–10.36)	0.000	16.53 (9.59–28.47)	**0.000**
Known diabetes						
No	775 (97)	2201 (>99)	1 (ref.)	–	1 (ref.)	**–**
Yes	64 (3)	9 (<1)	8.20 (3.83–17.59)	0.000	15.70 (6.54–37.71)	**0.000**
Other known pre-existing medical problems						
No	368 (46)	1777 (80)	1 (ref.)	–	–	–
Yes	433 (54)	433 (20)	4.83 (4.06–5.75)	0.000	7.39 (6.04–9.04)	**0.000**
**Pregnancy related characteristics**						
Parity						
0	308 (38)	926 (42)	1 (ref.)	–	–	**–**
1 to 2	358 (45)	1057 (48)	1.02 (0.85–1.21)	0.840	NA^b^	
≥3	128 (16)	227 (10)	1.70 (1.32–2.18)	0.000	NA^b^	
Missing	7 (1)	0				
Known multiple pregnancy						
No	777 (97)	2176 (98)	1 (ref.)	–	1 (ref.)	**–**
Yes	24 (3)	34 (2)	1.98 (1.17–3.35)	0.012	2.01 (1.05–3.84)	**0.035**
Known gestational diabetes						
No	763 (95)	2135 (97)	1 (ref.)	–	NA^b^	**–**
Yes	38 (5)	75 (3)	1.42 (0.95–2.11)	0.086	NA^b^	**–**

a Adjusted for Maternal age, Ethnic group, smoking in pregnancy, BMI, parity, known history of epilepsy, hypertension, cardiac disease, diabetes, other medical conditions, parity, known multiple pregnancy and gestational diabetes.

b The multivariable model showed that increasing parity and known gestational diabetes no longer contributed significantly to risk of maternal death and adjusted ORs for these factors are not shown.

Table 2 describes a more detailed examination of the association between maternal death and ethnic origin since disaggregate ethnic group routine population data were available for women who gave birth in 2018 and 2019. After adjustment for maternal age, social deprivation, multiple pregnancy and parity, women of African, Caribbean, and Other Black subgroups all remained at significantly higher risk of maternal death than White women. The effect was most marked in women of Caribbean and African ethnic origin (aOR 3.55 (2.30–5.48) and aOR 2.13 (1.60–2.84) respectively). Among women of Asian ethnic origin, only women of Indian ethnic origin were at a statistically significantly raised risk of maternal death after adjustment (aOR 1.51 (1.04–2.17)).

Since routine population data about women giving birth in the UK does not capture information about smoking status, body mass index (BMI), or pregnancy related or underlying medical conditions, Table 3 shows a comparison of the 801 women who died while pregnant or within the six weeks following the end of pregnancy with 2210 control women from the four UKOSS studies. In univariate analysis, women from non-white ethnic groups were at a significantly increased risk of maternal death (OR 1.79 (1.49–2.15)) with the risk most pronounced among women of Black aggregate ethnic origin (OR 3.15 (2.37–4.20)). Increasing maternal age, smoking in pregnancy, increasing BMI, known epilepsy, hypertension, cardiac disease, diabetes, or other underlying medical condition, increasing parity, and known multiple pregnancy were all significantly associated with increased risk of maternal death. Notably, women with known pre-existing medical conditions had a six-to-11-fold increased risk of maternal death depending upon the condition (epilepsy OR 8.45 (4.92–14.54); hypertension OR 11.51 (5.52–24.00), cardiac disease 6.20 (3.71–10.36) and diabetes 8.20 (3.83–17.59)). After adjusting for all these variables, women of Black and Asian aggregate ethnic origin remained at significantly increased risk of maternal death (OR 3.13 (2.21–4.43) and 1.57 (1.16–2.12) respectively).

**Fig. 2:**
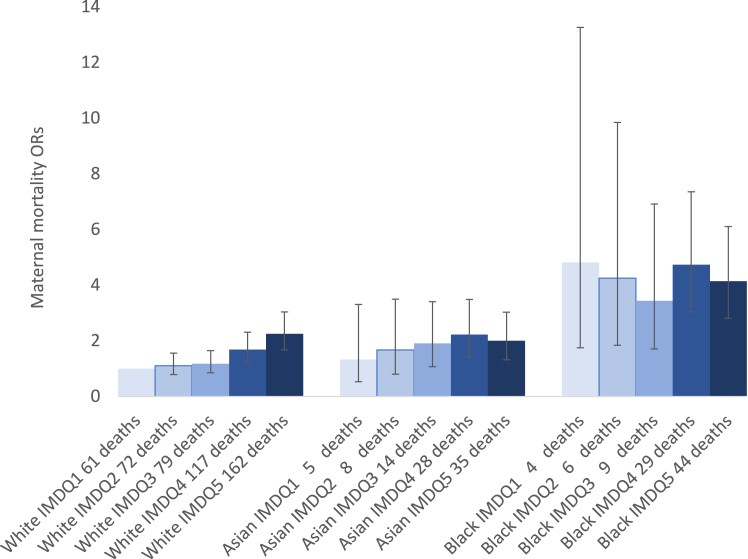
**Odds ratios (ORs) of maternal mortality for White, Asian, and Black women relative to the least deprived White women∗**. ∗Number of maternal deaths for each ethnicity and IMD quintile is shown on the X axis.

Among women of the White ethnic aggregate group, the risk of maternal mortality increased as IMD quintile increased. Women living in the most deprived IMD quintile had a significantly increased risk of maternal mortality compared with those in the least deprived (aOR 2.26, 95% CI 1.67–3.04, Fig. 2, Supplementary Table S2). However, this gradient was not observed across other ethnic groups, with no significant difference in the risk of maternal death between the IMD quintiles in women of Asian or Black ethnicity (Supplementary Tables S3 and S4). Women in the Black aggregate ethnic group had an increased risk of maternal mortality across all socioeconomic quintiles compared with women in the White aggregate group (Fig. 2).

## Discussion

This national population-based cohort analysis/study found that women in the Black aggregate ethnic group had a 3.2-fold increased risk of dying during pregnancy and 6 weeks after birth compared with women in the White aggregate ethnic group. After taking account of age, socioeconomic status and multiple pregnancy, this risk was still 2.4 times higher and after taking account of smoking, BMI, parity, pre-existing medical conditions, and diabetes the risk was still 3.1-fold higher than women in the White aggregate ethnic group. Women in the Asian aggregate ethnic group had a 34% increased risk of maternal death. After taking account of the socioeconomic status, this risk was no longer significantly increased for the group overall but remained elevated in women of Indian ethnicity. In women of White ethnicity we observed a socioeconomic gradient in mortality risk, whereas women of Black ethnicity were at increased risk irrespective of socioeconomic deprivation. Therefore, whilst these demographic, medical and pregnancy risk factors contribute to increased risk of maternal mortality in women of Black and Asian ethnic origin, they do not account for the overall increased risk found.

The findings of this national cohort are of relevance to other settings with observed ethnic disparities in maternal mortality. To our knowledge, only one other national study, which included all maternal deaths from 1979 to 1986 recorded through surveillance in the USA, has evaluated the contribution of known risk factors to ethnic disparities. They concluded that after adjustment for age, education, marital status, area of residence and antenatal care, women with Black ethnicity had a 3.53 (95% CI 2.9–4.4) fold increased risk of maternal death.^18^ Our study used data obtained through enhanced surveillance and is therefore at low risk of bias from missing deaths compared with much of the existing data which utilises registry data or modelled estimates.^2^^,^^4^ Therefore, whist the generalisability of the findings are dependent on population demographics, health service provision and social cultural factors in the UK, the policy and research implications of the key findings are likely generalisable across other high income settings.

This study adds to the literature, as the large number of women included mean there is sufficient statistical power to understand the risk to detailed ethnic groups, thus providing greater understanding of diversity of women affected.^12^ However, it is a limitation that in the routine population level data comparison with detailed ethnic groups was only available for two years of data. The latest census suggested that the proportions of ethnic minority groups is rapidly changing and this is not captured.^21^ In addition, a small number of women of Mixed ethnicity were excluded from this study (n = 14). Other studies undertaken in the UK have reported that women of Mixed ethnic origin were not at increased risk of severe maternal morbidity^14^ or mortality^27^ compared to women of White ethnicity, but many studies are limited by small numbers in this group, and future studies are warranted given this is a growing demographic.^21^

The use of multiple denominator data sources allowed the inclusion of a broad range of socioeconomic, medical and pregnancy related risks and the examination of intersectionality with socioeconomic deprivation between ethnic groups.^22^ ‘Ethnicity’ is used as a lens to examine inequity in outcomes, however it should not be considered an isolated exposure but as a dynamic, subjective social construct which can be an, often poor, proxy for interrelated socioeconomic aspects.^28^ In this study, despite the inclusion of multiple exposures, residual confounding from other factors that contribute to increased risk by ethnic groups is likely. Using the conceptual model proposed by Howell et al.^9^ and the WHO's Commission on Social Determinants of Health framework, other individual factors might include knowledge, beliefs, and health behaviours. Interventions or policies aiming to reduce inequity should therefore avoid modifying practice based on the construct of ethnicity alone, for example through inclusion in risk prediction models^29^ or guidelines,^30^ which may inadvertently exacerbate disparities, to tackling the underlying causes of disparities. For example, inadequate engagement with antenatal care has previously been demonstrated to partly contribute to disparities in mortality rates between ethnic groups in the UK.^10^ The reason for different engagement of services by ethnic group include challenges with access, such as language and cultural perception or experience of care.^31^ Whilst confidential enquiries into maternal death by different ethnic group in the UK did not identify any assessed difference in quality of care, multiple areas of bias such as lack of nuanced care and microaggressions differentially affected women from minority ethnic groups.^12^ This is in keeping with other studies which found evidence of dismissive care and fragmented services for women from minority ethnic groups.^32^^,^^33^ The development of culturally competent services to prevent implicit bias is frequently recommended, and a limited number of culturally adapted care or interventions to reduce inequalities relating to ethnicity exist.^9^^,^^34^ Racial bias exists not just at an interpersonal level but also an institutional level.^22^ For example, confidential enquiries in the UK have consistently reported that women who die have multiple social, physical, and mental health problems. Structural biases in the UK health system mean that current structures struggle to work across the boundary of health and social care services, meaning that women do not get high quality integrated services.^12^

We have shown women of Black ethnicity were at increased risk of maternal death across all deprivation levels compared with women of White ethnicity, with no trend in risk in relation to level of IMD. Index of Multiple Deprivation is a commonly used proxy for socioeconomic status and includes structural determinants of health: Income, education, occupation, and community factors such as crime and barriers to housing and services. A weighted mean across these domains is used to describe deprivation across small areas. This study included all births and maternal deaths across the countries of the UK and therefore utilised the IMD reference from each country, with differing measures within domains. Presentation of combined results for the UK means that the relative impact of deprivation between countries cannot be described. In addition, IMD does not describe the wider socioeconomic context such as societal values, wealth, isolation, and social capital, nor the intersection of racism and discrimination on the structural determinants of health. Evidence from the USA has shown that inequality in income across states is significantly associated with maternal mortality in women of Black but not White ethnicity. This suggests that it is not just deprivation of material resource that drives ethnic disparities in maternal mortality, but potentially the extent of the relative gap in distribution of income and resources.^35^ Therefore, whilst the strong association between pre-existing medical conditions and increased risk of mortality reinforces the need for nuanced, personalised care,^36^ policies aiming to reduce health disparities need to move beyond addressing individual factors but to equity in underlying structures such as housing, education and access to healthy environments.^3^

This study, the first national cohort of its kind in the UK, significantly enhances our understanding of the contribution of known risk factors to the increased risk of maternal death experienced by women of Black and Asian ethnicity living in the UK. Whilst age, BMI, socioeconomic status, and pre-existing medical complications are important risks for maternal mortality, we demonstrate that ethnicity remains an important independent risk, irrespective of these other factors. This, together with the observed differing impact of socioeconomic deprivation between ethnic groups, suggests that efforts to tackle implicit structural bias and deliver culturally competent maternity care that can mitigate against bias, are urgently required. Further research is required to understand the impact of structural change on ethnic inequalities, how to adapt and deliver culturally competent services, and how to implement this change within the current constraints of overburdened maternity services.

## Contributors

KB, SK, JK, NV and MK conceptualised and designed the study. KB, MK and NV had access to and verified the data reported in the manuscript, and undertook data analysis. All authors contributed to data interpretation. NV and KB wrote original draft and generated figures and tables. SK, JK and MK contributed to review and editing.

## Data sharing statement

Data may be requested through the Healthcare Quality Improvement Partnership: https://www.hqip.org.uk/national-programmes/accessing-ncapop-data/.

## Declaration of interests

All authors have completed the ICMJE disclosure form and declare: MK, SK and JK received grants from the NIHR Policy Research Programme in relation to the submitted work. JK, MK and SK are part of the MBRRACE-UK Collaboration. KB and NV have no financial relationships with any organisations that might have an interest in the submitted work in the previous three years. No authors have other relationships or activities that could appear to have influenced the submitted work. The views expressed are those of the author(s) and not necessarily those of the NIHR or the Department of Health and Social Care.
