# Supplementary material for: Impact of maternal risk factors on ethnic disparities in maternal mortality: a national population-based cohort study
Source: Lancet Reg Health Eur. 2024 Mar 29;40:100893. doi: 10.1016/j.lanepe.2024.100893 (PMC10998184; doi:10.1016/j.lanepe.2024.100893)
Supplement: Supplementary Tables [file mmc1.docx]

**Supplementary Table 1: Ethnic categories in the 2021 census classification for England and Wales***

| Asian, Asian British or Asian Welsh | Bangladeshi |
| --- | --- |
|  | Chinese |
|  | Indian |
|  | Pakistani |
|  | Other Asian |
| Black, Black British, Black Welse, Caribbean or African | African |
|  | Caribbean |
|  | Other Black |
| Mixed or Multiple ethnic groups | White and Asian |
|  | White and Black African |
|  | White and Black Caribbean |
|  | Other Mixed or Multiple ethnic Groups |
| White | Irish |
|  | Gypsy or Irish Traveller |
|  | Roma |
|  | White: Englsh, Welsh, Scottish, Northern Irish or British |
|  | Other White |
| Other ethnic group | Arab |
|  | Any other ethnic group |

*In keeping with the census reporting we have presented ethnicity alphabetically for the aggregate groups and 19 ethnic categories with “Other” options listed last.

**Supplementary Figure 1: Directed Acyclic Graph (DAG) to conceptually represent association between covariates and unmeasured factors. Informed by existing**

**literature and clinical knowledge.**


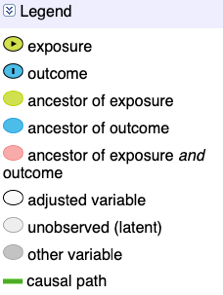

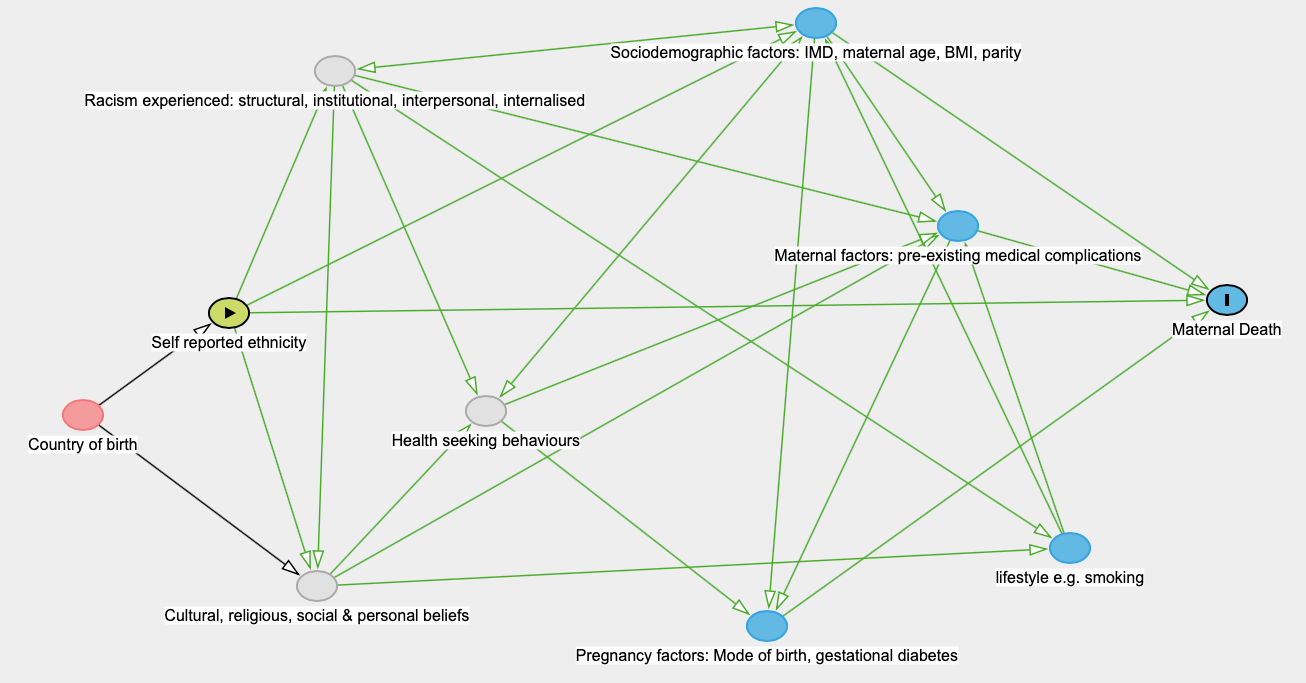


**Supplementary Table 2: Risk of maternal mortality across Index of Multiple Deprivation quintiles in women of Black and Asian ethnicity compared to women of White ethnicity.**

| Index of Multiple Deprivation* | White aggregate ethnic group**  aOR (95% CI) | Black Aggregate ethnic Group  aOR (95% CI) | Asian Aggregate ethnic group  aOR (95% CI) |
| --- | --- | --- | --- |
| IMD Quartile 1 | 1 | 4.82 (1.75-8.45) | 1.33 (0.53-3.31) |
| IMD Quartile 2 | 1.11 (0.79-1.56) | 4.26 (1.84-5.59) | 1.65 (0.80-3.50) |
| IMD Quartile 3 | 1.18 (0.85-1.65) | 3.44 (1.71-6.92) | 1.91 (1.07-3.41) |
| IMD Quartile 4 | 1.69 (1.24-2.31) | 4.73 (3.04-7.36) | 2.23 (1.42-3.49) |
| IMD Quartile 5 | 2.26 (1.67-3.04) | 4.14 (2.81-6.11) | 2.00 (1.32-3.03) |

* Index of Multiple Deprivation is a commonly used proxy for socioeconomic status and includes structural determinants of health: Income, education, occupation, and community factors such as crime and barriers to housing and services. It is calculated using routine data to produce a weighted mean across these domains to describe deprivation across small areas.

** ethnicity data was available as aggregated, or high level, groupings used in the census classification

**Supplementary Table 3: Risk of maternal mortality across Index of Multiple Deprivation quintiles in women of White and Asian ethnicity compared to women of Black ethnicity.**

| Index of Multiple Deprivation* | White aggregate ethnic group  aOR (95% CI)** | Black Aggregate ethnic Group  aOR (95% CI) | Asian Aggregate ethnic group  aOR (95% CI) |
| --- | --- | --- | --- |
| IMD Quartile 1 | 0.21 (0.07-0.57) | 1 | 0.28 (0.07-1.02) |
| IMD Quartile 2 | 0.23 (0.08-0.63) | 0.88 (0.24-3.13) | 0.35 (0.10-1.15) |
| IMD Quartile 3 | 0.25 (0.09-0.67) | 0.71 (0.22-2.31) | 0.40 (0.13-2.31) |
| IMD Quartile 4 | 0.35 (0.13-0.95) | 0.98 (0.34-2.79) | 0.46 (0.16-1.20) |
| IMD Quartile 5 | 0.47 (0.17-0.95) | 0.86 (0.31-2.39) | 0.41 (0.15-1.17) |

* Index of Multiple Deprivation is a commonly used proxy for socioeconomic status and includes structural determinants of health: Income, education, occupation, and community factors such as crime and barriers to housing and services. It is calculated using routine data to produce a weighted mean across these domains to describe deprivation across small areas.

** ethnicity data was available as aggregated, or high level, groupings used in the census classification

**Supplementary Table 4: Risk of maternal mortality across Index of Multiple Deprivation quintiles in women of White and Black ethnicity compared to women of Asian ethnicity.**

| Index of Multiple Deprivation* | White aggregate ethnic group  aOR (95% CI)** | Black Aggregate ethnic Group  aOR (95% CI) | Asian Aggregate ethnic group  aOR (95% CI) |
| --- | --- | --- | --- |
| IMD Quartile 1 | 0.75 (0.30-1.87) | 3.63 (0.97-13.52) | 1 |
| IMD Quartile 2 | 0.83 (0.33-2.06) | 3.20 (0.98-10.50) | 1.26 (0.41-3.86) |
| IMD Quartile 3 | 0.89 (0.36-2.20) | 2.59 (0.87-7.72) | 1.43 (0.52-3.98) |
| IMD Quartile 4 | 1.27 (0.52-3.11) | 3.56 (1.38-9.20) | 1.68 (0.65-4.35) |
| IMD Quartile 5 | 1.70 (0.70-4.14) | 3.12 (1.24-7.87) | 1.50 (0.59-3.84) |

* Index of Multiple Deprivation is a commonly used proxy for socioeconomic status and includes structural determinants of health: Income, education, occupation, and community factors such as crime and barriers to housing and services. It is calculated using routine data to produce a weighted mean across these domains to describe deprivation across small areas.

** ethnicity data was available as aggregated, or high level, groupings used in the census classification

STROBE Statement

|  | Item No | Recommendation |  |
| --- | --- | --- | --- |
| Title and abstract | 1 | (a) Indicate the study’s design with a commonly used term in the title or the abstract | Page 1, line 1 |
|  |  | (b) Provide in the abstract an informative and balanced summary of what was done and what was found | Page 2 |
| Introduction | | |  |
| Background/rationale | 2 | Explain the scientific background and rationale for the investigation being reported | Page 3 from Line 11 |
| Objectives | 3 | State specific objectives, including any prespecified hypotheses | Page 6, line 18 |
| Methods | | |  |
| Study design | 4 | Present key elements of study design early in the paper | Page 9 from line 13 |
| Setting | 5 | Describe the setting, locations, and relevant dates, including periods of recruitment, exposure, follow-up, and data collection | Page 7, line 1-3 |
| Participants | 6 | (a) Give the eligibility criteria, and the sources and methods of selection of participants. Describe methods of follow-up | Page 7 paragaph 1 |
|  |  | (b) For matched studies, give matching criteria and number of exposed and unexposed | NA |
| Variables | 7 | Clearly define all outcomes, exposures, predictors, potential confounders, and effect modifiers. Give diagnostic criteria, if applicable | Page 9, from line 13 |
| Data sources/ measurement | 8* | For each variable of interest, give sources of data and details of methods of assessment (measurement). Describe comparability of assessment methods if there is more than one group | Page 7 and 8 |
| Bias | 9 | Describe any efforts to address potential sources of bias |  |
| Study size | 10 | Explain how the study size was arrived at | Page 9, line 20-21 |
| Quantitative variables | 11 | Explain how quantitative variables were handled in the analyses. If applicable, describe which groupings were chosen and why | Page 10, line 1 and 21 |
| Statistical methods | 12 | (a) Describe all statistical methods, including those used to control for confounding | Page 9, line 13 |
|  |  | (b) Describe any methods used to examine subgroups and interactions | NA |
|  |  | (c) Explain how missing data were addressed | Page 10 line 25 to page 11 line 3 |
|  |  | (d) If applicable, explain how loss to follow-up was addressed | NA |
|  |  | (e) Describe any sensitivity analyses | NA |
| Results | | |  |
| Participants | 13* | (a) Report numbers of individuals at each stage of study—eg numbers potentially eligible, examined for eligibility, confirmed eligible, included in the study, completing follow-up, and analysed | Figure 1, page 11, line 11-14 |
|  |  | (b) Give reasons for non-participation at each stage | NA |
|  |  | (c) Consider use of a flow diagram | Figure 1 |
| Descriptive data | 14* | (a) Give characteristics of study participants (eg demographic, clinical, social) and information on exposures and potential confounders | Table 1 |
|  |  | (b) Indicate number of participants with missing data for each variable of interest | Tables |
|  |  | (c) Summarise follow-up time (eg, average and total amount) | NA |
| Outcome data | 15* | Report numbers of outcome events or summary measures over time | page 11, line 11-14 |
| Main results | 16 | (a) Give unadjusted estimates and, if applicable, confounder-adjusted estimates and their precision (eg, 95% confidence interval). Make clear which confounders were adjusted for and why they were included | Table 1-3 and results text |
|  |  | (b) Report category boundaries when continuous variables were categorized | Table 1 |
|  |  | (c) If relevant, consider translating estimates of relative risk into absolute risk for a meaningful time period | NA |
| Other analyses | 17 | Report other analyses done—eg analyses of subgroups and interactions, and sensitivity analyses | NA |
| Discussion | | |  |
| Key results | 18 | Summarise key results with reference to study objectives | Page 13, 18-25 |
| Limitations | 19 | Discuss limitations of the study, taking into account sources of potential bias or imprecision. Discuss both direction and magnitude of any potential bias | Interpretation results 3-5 |
| Interpretation | 20 | Give a cautious overall interpretation of results considering objectives, limitations, multiplicity of analyses, results from similar studies, and other relevant evidence | Page 17, paragraph 1 |
| Generalisability | 21 | Discuss the generalisability (external validity) of the study results | Page 14, line 13-16 |
| Other information | | |  |
| Funding | 22 | Give the source of funding and the role of the funders for the present study and, if applicable, for the original study on which the present article is based | Page 17, line 22 onwards |
